# Supplementary material for: Long distance entanglement and high-dimensional quantum teleportation in the Fermi–Hubbard model
Source: Sci Rep. 2023 Jan 18;13:964. doi: 10.1038/s41598-023-28180-4 (PMC9849208; doi:10.1038/s41598-023-28180-4)
Supplement: Supplementary file 1 — Supplementary Figures. [file 41598_2023_28180_MOESM1_ESM.pdf]

Supplementary Material

1 Standard Teleportation Protocol

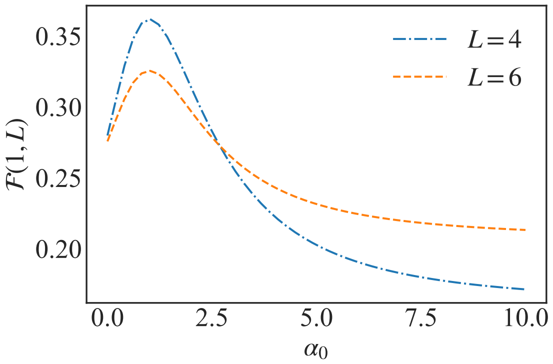

Figure S1: The end-to-end fidelity of the standard quantum teleportation channel as a function of the coefficient α_0 for various system's sizes L and $U = 0$, using Bell state projective measurements.

2 Teleportation protocol with Hubbard Projective Measurements

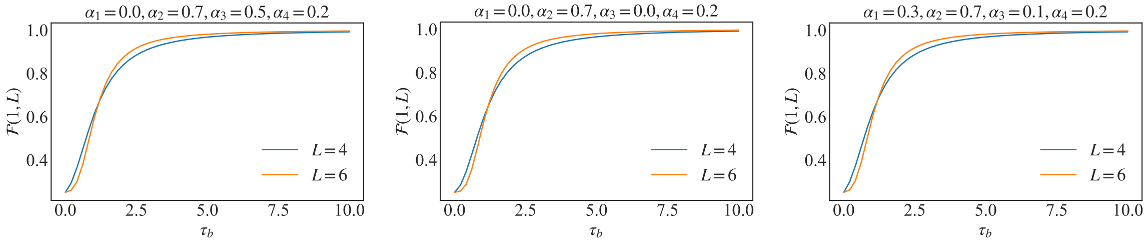

(a) (b) (c)

Figure S2: The end-to-end fidelity of the standard quantum teleportation protocol using Hubbard projective measurements, for different values of the coefficients α_i, with $i = 0, 1, 2, 3$ and various sizes L.
